# Comparative Analysis of ISM-1 and B7-H3 Expression in Castration-Resistant Prostate Adenocarcinoma: Associations with Tumor Aggressiveness and Resistance Dynamics

**DOI:** 10.3390/medicina62030477

**Published:** 2026-03-03

**Authors:** Şeyhmus Kaya, Abuzer Öztürk, Ramazan Oğuz Yüceer, Nisa Begüm Öztürk

**Affiliations:** 1 Department of Pathology, Faculty of Medicine, Sivas Cumhuriyet University, 58140 Sivas, Turkey; r.yuceer66@hotmail.com (R.O.Y.); nb_unluer@hotmail.com (N.B.Ö.); 2 Department of Urology, Sivas Numune Hospital, 58140 Sivas, Turkey; brusksidal@gmail.com

**Keywords:** castration-resistant prostate cancer, ISM-1, B7-H3, immunohistochemistry

## Abstract

*Background and Objectives*: Prostate cancer exhibits substantial biological heterogeneity. Although several biomarkers reflecting aggressive tumor behavior have been identified, molecular indicators related to biological adaptation to androgen deprivation remain limited. This study aimed to comparatively evaluate ISM-1 and B7-H3 expression in localized prostate cancer (LPC) and the pre-CRPC group (pre-treatment diagnostic biopsy tissue from patients who subsequently developed CRPC), and to investigate their clinicopathological associations in the pre-CRPC group. *Materials and Methods*: This retrospective study included 30 surgically treated LPC cases and 32 pre-CRPC cases with available prostate tissue samples obtained prior to the development of castration resistance. ISM-1 and B7-H3 expression levels were evaluated immunohistochemically using the H-score method (intensity 0–3 × proportion score 0–3 [0%, 1–19%, 20–50%, >50%]). Expression patterns were compared between LPC and pre-CRPC groups. Within the pre-CRPC group, associations with clinicopathological parameters were evaluated using H-scores as continuous variables, and time to castration resistance was analyzed using Cox regression. *Results*: ISM-1 expression was generally low in LPC cases, with a median H-score of 0, whereas pre-CRPC biopsy tissues demonstrated a marked increase in ISM-1 expression. B7-H3 expression was higher and more homogeneous in the pre-CRPC group compared with LPC. In the pre-CRPC group, ISM-1 and B7-H3 H-scores showed a strong positive correlation. No statistically significant associations were identified between ISM-1 or B7-H3 expression levels and most conventional clinicopathological parameters; however, both markers differed significantly across Grade Groups. Neither marker showed a statistically significant association with time to castration resistance, although ISM-1 demonstrated a non-significant trend toward a longer time to resistance. *Conclusions*: ISM-1 and B7-H3 exhibit distinct expression patterns across different stages of prostate cancer but show associated expression profiles in the pre-CRPC group. While B7-H3 appears to reflect aggressive tumor biology, the independent expression pattern of ISM-1 and its non-significant trend toward a longer time to resistance suggest a potential role in the clinical trajectory toward castration resistance. Combined assessment of ISM-1 and B7-H3 may contribute to a better understanding of tumor biology in patients who subsequently develop CRPC. These findings are descriptive and hypothesis-generating.

## 1. Introduction

Prostate cancer (PCa) is one of the most frequently diagnosed non-cutaneous malignancies among men in Western countries, with an estimated lifetime risk of approximately one in nine men. It represents the third leading cause of cancer-related mortality, accounting for 11.3% of cancer deaths in Europe. The clinical course of PCa is notably heterogeneous. While the disease may remain organ-confined for prolonged periods in some patients, others develop progressive and treatment-resistant disease. The main therapeutic strategies include surgery, radiotherapy, androgen deprivation therapy (ADT), and chemotherapy. Although high survival rates can be achieved in localized prostate cancer (LPC) with surgery and radiotherapy, ADT—despite its initial effectiveness in advanced disease—ultimately leads to the development of castration-resistant prostate cancer (CRPC) in a substantial proportion of patients. CRPC represents the most challenging stage of prostate cancer due to limited therapeutic options and poor clinical prognosis, with an average survival of 2–3 years in advanced disease [1,2,3]. Therefore, accurate prediction of tumor biological behavior during progression to castration resistance has become increasingly important. Conventional clinical and histopathological parameters, such as serum prostate-specific antigen (PSA) levels, tumor grade, and metastatic burden, are insufficient to fully reflect disease biology and adaptive mechanisms in CRPC. This limitation highlights the urgent need for novel molecular biomarkers capable of better characterizing CRPC biology and predicting disease behavior.

B7-H3 (CD276) is a member of the B7 family that functions as an immune regulatory checkpoint molecule and was first described in 2001 [4]. It has been shown to be highly expressed in a wide range of solid tumors, including lung, breast, ovarian, head and neck, and gastrointestinal malignancies, and has been reported to be associated with tumor aggressiveness, metastatic potential, treatment resistance, and poor prognosis [5,6]. In prostate cancer, B7-H3 is strongly expressed in tumor epithelium and has been linked to higher tumor grade, advanced disease stage, and unfavorable clinical outcomes, suggesting that it may be associated with aggressive tumor biology [7,8].

Isthmin-1 (ISM-1) was initially identified in *Xenopus* embryos in 2002 and was later characterized in 2021 as an adipose tissue-derived (predominantly secreted) protein expressed in multiple tissues, with pleiotropic biological functions related to cellular homeostasis. The *ISM-1* gene, a member of the isthmin gene family located on chromosome 20, encodes proteins containing thrombospondin type 1 repeat and AMOP domains. ISM-1 has been implicated in diverse pathophysiological processes, including metabolism, immune regulation, tumor development, cell growth, endothelial permeability, and other physiological functions [9]. In recent years, increasing interest has focused on ISM-1 in the context of tumor biology, with studies reporting heterogeneous expression patterns in tumor cells and the tumor microenvironment across various solid malignancies. These findings suggest that ISM-1 may be involved not only in physiological processes but also in tumor progression and cellular adaptation to environmental stress. However, in prostate cancer—particularly in tumors from patients who later develop CRPC (pre-resistance diagnostic tissue)—the immunohistochemical expression profile of ISM-1 and its clinicopathological implications remain largely unexplored [10].

The aim of this study was to comparatively evaluate the immunohistochemical expression of ISM-1 and B7-H3 in localized prostate adenocarcinoma pre-CRPC diagnostic biopsy tissue obtained prior to ADT from patients who subsequently developed CRPC, and to explore their associations with clinical and pathological parameters, particularly within the pre-CRPC group. This exploratory, hypothesis-generating approach may contribute to a better understanding of the baseline ISM-1 expression patterns in prostate cancer and explore the potential clinical relevance of its combined assessment with B7-H3.

## 2. Materials and Methods

### 2.1. Study Design and Population

This retrospective study was conducted at the Department of Pathology, Faculty of Medicine, Sivas Cumhuriyet University. A total of 62 patients diagnosed with prostatic acinar adenocarcinoma between 1 January 2014 and 31 December 2022 were included in the study. The study sample comprised two groups: the first consisted of 30 cases with localized prostate cancer who underwent radical prostatectomy. The second group comprised 32 patients who subsequently developed CRPC and had available pre-CRPC biopsy tissue obtained from tru-cut biopsies collected at the time of initial diagnosis. These biopsies represent tissue samples obtained prior to the initiation of ADT.

Demographic data (age) and baseline clinical and pathological variables—including serum prostate-specific antigen (PSA) levels, tumor Grade Group, and the presence of comorbidities—were retrospectively obtained for both case groups from hospital electronic medical records and final pathology reports. In the group comprising cases that subsequently developed CRPC, clinically relevant variables—namely perineural invasion (PNI), seminal vesicle invasion (SVI), lymph node metastasis (LNM), bone metastasis (BM), applied treatment modalities (ADT, chemotherapy, and radiotherapy), and time to progression to castration resistance—were recorded and analyzed exclusively for this study group. The presence of metastasis was assessed in accordance with the retrospective design of the study using imaging modalities available at the institution during the relevant period. Staging imaging was performed at the time of the prostate biopsy procedure or prior to initiation of treatment, and evaluations for lymph node and bone metastases were conducted at the same clinical time point. Lymph node involvement was evaluated using contrast-enhanced computed tomography and/or magnetic resonance imaging. Bone metastases were investigated using technetium-99 m bone scintigraphy; additionally, positron emission tomography-computed tomography and/or prostate-specific membrane antigen positron emission tomography were utilized in selected cases. Histopathological diagnoses of all cases were independently confirmed by two experienced pathologists. Pathological variables, including tumor Grade Group, perineural invasion, seminal vesicle invasion, lymph node metastasis, and bone metastasis, were defined as categorical variables and evaluated as present/absent or according to appropriate subgroups. Clinical treatment-related variables were categorized as applied or not applied and included in the analysis accordingly. Cases with histopathologically confirmed diagnoses, complete clinical and pathological data, and sufficient formalin-fixed paraffin-embedded tissue available for immunohistochemical analysis were included in the study. Cases lacking diagnostic certainty, those with incomplete data, or specimens containing artifacts severe enough to preclude reliable evaluation were excluded. Consequently, a total of seven cases were excluded from the final analysis.

Only cases that underwent radical prostatectomy were included in the LPC group. This group was composed of Grade Group 1 cases in order to represent organ-confined disease with low risk. However, because the Grade Group distribution differed between the groups, the observed expression differences may be partly related to tumor grade and should be taken into account when interpreting the findings. As prostatectomy specimens were not available for cases that subsequently developed CRPC, the evaluation in this group was performed on diagnostic biopsy samples. These biopsies were obtained prior to the initiation of ADT and are referred to in this study as pre-CRPC biopsy tissue.

The primary endpoint of this study was to compare the immunoexpression levels of ISM-1 and B7-H3 between LPC tissue and pre-CRPC tissues from cases that subsequently developed CRPC. H-scores were calculated for both markers, and differences between the groups were evaluated accordingly. The secondary endpoints comprised the assessment of associations between ISM-1 and B7-H3 H-scores and age, serum PSA level, tumor Grade Group, PNI, SVI, LNM and BM, and applied treatment modalities (chemotherapy and radiotherapy) in cases that subsequently developed CRPC. In addition, the relationship between marker levels and time to development of castration resistance was evaluated, and the correlation between these expression levels was also examined as part of the secondary analyses.

### 2.2. Histopathological and Immunohistochemical Evaluation

Hematoxylin and eosin-stained slides from all cases were retrieved from the pathology archives and re-evaluated. The diagnosis of prostatic acinar adenocarcinoma was confirmed according to the World Health Organization (2022) classification criteria. During histopathological evaluation, relevant pathological parameters—including tumor Grade Group, perineural invasion, seminal vesicle invasion, and metastatic status—were recorded. All assessments were performed independently by two experienced pathologists who were blinded to clinical and prognostic data. In cases of discordance, a consensus diagnosis was reached through joint review.

For immunohistochemical analysis, 4-µm thick sections were obtained from formalin-fixed, paraffin-embedded tissue blocks. Immunostaining was performed using a fully automated Ventana BenchMark system. On-board deparaffinization, rehydration, and heat-induced antigen retrieval were carried out according to the manufacturer’s standard protocols. Following endogenous peroxidase blocking, immunoreactivity was visualized using a diaminobenzidine-based detection system, and sections were counterstained with hematoxylin.

Immunohistochemical staining for ISM-1 was performed using a polyclonal anti–ISM-1 primary antibody at a (Invitrogen, Thermo Fisher Scientific, Carlsbad, CA, USA; Cat. No. PA5-24968) dilution of 1:50, incubated for 44 min at 37 °C. The antibody was obtained from Invitrogen. Immunohistochemical staining for B7-H3 was performed using a monoclonal anti-CD276 antibody at a dilution of 1:4000, in accordance with the manufacturer’s recommendations. The antibody used was clone EPR20115 and was supplied by Abcam (Cambridge, UK). Immunohistochemical evaluation was restricted to tumor epithelial cells. ISM-1 expression was assessed based on nuclear staining, whereas B7-H3 expression was evaluated according to membranous and/or cytoplasmic staining patterns. Stromal staining and nonspecific background reactions were excluded from the analysis. Staining was scored semi-quantitatively based on staining intensity and the proportion of positive tumor cells. Staining intensity was graded as 0 (negative), 1 (weak), 2 (moderate), or 3 (strong), while the proportion of positive cells was scored as 0 (0%), 1 (1–19%), 2 (20–50%), or 3 (>50%). For each specimen, the H-score was derived by multiplying the staining intensity grade by the corresponding proportion score. The resulting H-scores were used as continuous variables in the statistical analyses for both the LPC and pre-CRPC biopsy tissue groups.

Appropriate positive and negative controls were included for the immunohistochemical procedures. Tonsillar tissue was used as the positive control for both ISM-1 and B7-H3 immunostaining. Negative controls were prepared by omitting the primary antibody from the staining protocol. The evaluation was restricted to tumor epithelial cells; stromal/inflammatory cell staining was considered background reactivity and was excluded from scoring. Interpretation of staining patterns was guided by the manufacturers’ datasheets and findings reported in the literature [11].

### 2.3. Statistical Analysis

All statistical evaluations were carried out using SPSS software for Windows (version 23.0; SPSS Inc., Chicago, IL, USA). Continuous variables were summarized as median (interquartile range, IQR) and categorical variables as number (%). Between-group comparisons (LPC vs. pre-CRPC) were conducted using the Mann–Whitney U test for continuous/ordinal variables and the chi-square test or Fisher’s exact test for categorical variables, as appropriate. For within–pre-CRPC analyses, ISM-1 and B7-H3 H-scores were analyzed as continuous variables. Comparisons of H-scores across binary clinicopathological variables were performed using the Mann–Whitney U test, while comparisons across multi-category variables (e.g., Grade Group) were assessed using the Kruskal–Wallis test followed by Dunn’s post hoc test when an overall difference was detected. Effect sizes for two-group comparisons were quantified using Cliff’s delta (δ) [12] and interpreted as negligible (<0.147), small (0.147–0.330), medium (0.330–0.474), or large (>0.474). Associations between H-scores and continuous clinical variables (age and serum PSA) were evaluated using Spearman’s rank correlation. Time to development of castration resistance (months) was analyzed using Cox proportional hazards regression, with ISM-1 and B7-H3 H-scores entered as continuous covariates; hazard ratios (HRs) with 95% confidence intervals (CIs) were reported. All tests were two-sided, and *p* < 0.05 was considered statistically significant.

## 3. Results

**Figure 1:**
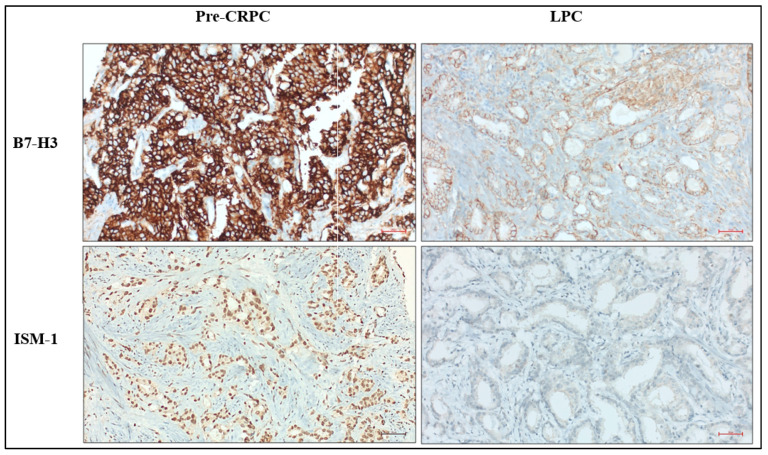
Representative immunohistochemical staining of ISM-1 and B7-H3 in localized prostate cancer (LPC) cases and in pre-CRPC biopsy tissue from cases that subsequently developed castration-resistant prostate cancer (CRPC). ISM-1 shows nuclear staining (immunoperoxidase, ×200), whereas B7-H3 exhibits a membranous and/or cytoplasmic staining pattern (immunoperoxidase, ×200). Scale bar: 50 µm in all panels.

**Figure 2:**
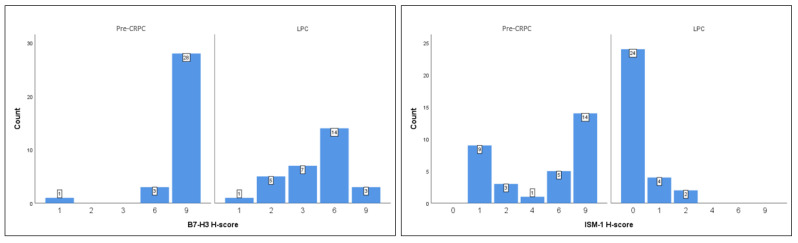
Histograms showing the distribution of B7-H3 and ISM-1 H-scores in LPC and pre-CRPC cases ((**left panel**): B7-H3 H-score; (**right panel**): ISM-1 H-score). Bars represent the number of cases at each H-score level.

A total of 62 cases of prostatic acinar adenocarcinoma were included in this study, comprising 32 cases who subsequently developed CRPC (pre-CRPC biopsy tissue) and 30 cases with LPC. The mean age of patients in the CRPC group (patients who subsequently developed CRPC) was 73.16 ± 8.43 years (range: 56–91), whereas the mean age of patients in the LPC group was 63.53 ± 6.39 years (range: 43–72). The clinical, demographic, and immunohistochemical characteristics of both groups are summarized in Table 1. Patients in the pre-CRPC group were significantly older than those in the LPC group and had significantly higher serum PSA levels. In addition, clinically significant comorbidities were more frequently observed among the pre-CRPC group. Immunohistochemical evaluation revealed that ISM-1 expression was generally low in LPC cases, with a median H-score of 0; however, weak or focal ISM-1 positivity was observed in a limited number of LPC cases. In contrast, ISM-1 expression was significantly increased in pre-CRPC group. Similarly, B7-H3 expression was significantly higher in the pre-CRPC group compared with the LPC group (Figure 1 and Figure 2). Differences in ISM-1 and B7-H3 expression levels between the CRPC (pre-CRPC biopsy tissue) and LPC groups were statistically significant.

The clinicopathological characteristics of the pre-CRPC group (patients who subsequently developed CRPC) are presented in detail in Table 2. The majority of cases in the pre-CRPC group consisted of high-grade tumors (Grade Groups 4 and 5). Within this cohort, the presence of SVI, PNI, LNM, and BM was recorded. All patients in the pre-CRPC group received ADT, while a subset of cases also underwent radiotherapy and/or chemotherapy. The median time from diagnosis to the development of castration resistance was 12 months.

Table 3 summarizes the associations between ISM-1 and B7-H3 H-scores and clinicopathological variables in the pre-CRPC cohort. ISM-1 H-scores differed significantly across Grade Group categories (*p* < 0.001). In Dunn post hoc analyses for multiple comparisons, this difference was mainly driven by comparisons between Grade Group 2 and Grade Groups 3, 4, and 5 (*p* = 0.001, *p* = 0.003, and *p* < 0.001, respectively). No statistically significant differences in ISM-1 H-scores were observed for the remaining variables included in the table (all *p* > 0.05). However, when effect sizes were examined using Cliff’s δ, negligible effects were observed for comorbidity and chemotherapy, small effects for SVI, PNI, BM, and radiotherapy, and a medium effect for lymph node metastasis.

Similarly, B7-H3 H-scores also differed significantly across Grade Group categories (*p* < 0.001), and Dunn post hoc analyses indicated that the overall difference was attributable to comparisons between Grade Group 2 and Grade Groups 3, 4, and 5 (*p* = 0.015, *p* = 0.003, and *p* < 0.001, respectively). No statistically significant differences were detected for the other variables (all *p* > 0.05). Based on Cliff’s δ, negligible effects were noted for comorbidity, SVI, PNI, BM, and radiotherapy, whereas small effects were observed for LNM and chemotherapy.

Taken together, these findings suggest that, given the limited sample size, *p*-values alone may be insufficient and potentially misleading; therefore, considering effect sizes alongside statistical significance may provide a more balanced appraisal of the potential clinicopathological associations of ISM-1 and B7-H3.

In cases that subsequently developed CRPC, the correlations of ISM-1 and B7-H3 H-scores with age and serum PSA levels were evaluated using Spearman’s rank correlation analysis. No statistically significant correlation was observed between ISM-1 H-scores and age (r = −0.068; *p* = 0.713) or PSA (r = −0.038; *p* = 0.835). Similarly, B7-H3 H-scores showed no statistically significant association with PSA (r = −0.036; *p* = 0.845) or age (r = −0.340; *p* = 0.057).

A Cox proportional hazards regression analysis was performed to identify factors associated with the time to development of castration resistance, and ISM-1 and B7-H3 H-scores were entered into the model as continuous variables. The omnibus tests of model coefficients did not reach statistical significance (*p* = 0.516). A negative trend was observed between the ISM-1 H-score and the risk of developing castration resistance (B = −0.063). Accordingly, the hazard ratio (HR) for ISM-1 was 0.939 (95% CI: 0.845–1.044; *p* = 0.244), suggesting that each one-unit increase in the ISM-1 H-score may be numerically associated with an approximately 6.1% decrease in the risk of developing castration resistance; however, this association was not statistically significant. Similarly, the HR for the B7-H3 H-score was 1.063 (95% CI: 0.841–1.343; *p* = 0.609), indicating no statistically significant effect on time to castration resistance (Table 4).

**Figure 3:**
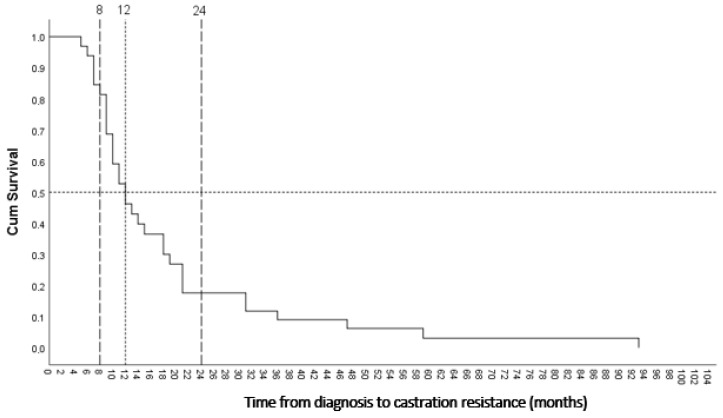
Curve depicting the time from diagnosis to development of castration resistance in the pre-CRPC group. The median time to castration resistance was 12 months, and castration resistance developed between 8 and 24 months in a substantial proportion of cases. The subsequent flattening of the curve after 24 months indicates that, in some cases, the time to castration resistance may be prolonged.

In the Cox proportional hazards regression analysis, the covariate means were calculated as ISM1_H = 5.469 and B7H3_H = 8.469. In Figure 3, the time point at which the cumulative survival for remaining castration-sensitive decreases to 0.5 (50%) corresponds exactly to 12 months. This finding indicates that the likelihood of developing castration resistance becomes evident at approximately 12 months after diagnosis. The steepest segment of the curve lies between 8 and 24 months, and the stepwise declines suggest that the majority of cases transition to castration resistance during this critical two-year period. After 24 months, the curve flattens, demonstrating that in some cases, a prolonged castration-sensitive course may be observed, extending up to 90 months.

When B7-H3 expression was evaluated, the majority of pre-CRPC cases were categorized as having high expression, while cases with low expression were limited in number. Correlation analysis revealed a strong and positive statistically significant association between ISM-1 and B7-H3 H-scores in pre-CRPC cases (Table 5). This finding indicates that both biomarkers exhibit a concurrent and interrelated expression profile in castration-resistant prostate cancer.

To summarize the leading findings, in the comparison of 30 LPC cases and 32 cases that subsequently developed CRPC, the group that later progressed to CRPC had significantly higher age, serum PSA levels, and comorbidity frequency than the LPC group. ISM-1 and B7-H3 H-scores were significantly higher in pre-CRPC biopsy tissue compared with LPC. In the group that subsequently developed CRPC, the median time from diagnosis to the development of castration resistance was 12 months, and resistance emerged in a substantial proportion of cases within 8–24 months. In the clinicopathological association analyses, both ISM-1 and B7-H3 H-scores differed significantly across Grade Groups; the primary source of this difference was the comparison between GG2 and GG3–5. However, no significant differences were detected with other variables, and effect sizes (Cliff’s δ) were mostly in the negligible/small range. There was a strong positive correlation between ISM-1 and B7-H3 H-scores, whereas no significant correlations were found with age or PSA. In Cox regression analysis, the biomarkers were not significantly associated with time to castration resistance, although a negative trend was observed for ISM-1.

## 4. Discussion

In this study, we compared the immunohistochemical expression of ISM-1 and B7-H3 between the LPC group and the pre-CRPC group consisting of patients who later developed CRPC. Both biomarkers showed higher H-scores in the pre-CRPC biopsies, and their expression levels co-varied strongly, suggesting a concurrent expression pattern. Within the pre-CRPC group, ISM-1 and B7-H3 H-scores differed significantly across Grade Groups, whereas associations with other clinicopathological variables were largely non-significant and effect sizes were mostly negligible-to-small. Neither biomarker was significantly associated with time to castration resistance; however, ISM-1 showed a non-significant trend toward lower risk. Overall, these findings suggest that elevated baseline ISM-1 and B7-H3 expression may be associated with a higher-grade tumor phenotype present before ADT; however, inferences regarding resistance biology should remain hypothesis-generating given group imbalances and tissue-type differences.

B7-H3 is overexpressed by tumor cells in a wide range of solid malignancies and has been consistently associated with adverse clinical outcomes. In the prostate cancer specific literature, increased B7-H3 expression has been linked to higher tumor grade, advanced disease stage, and metastatic spread [1,2]. In one study, B7-H3 expression was investigated in prostate biopsy specimens obtained during the hormone-sensitive and hormone-resistant phases of CRPC, with positivity rates of 97% and 93%, respectively. Moreover, previous reports have demonstrated that B7-H3 expression is significantly higher in metastatic prostate carcinoma compared with localized disease [5]. ADT represents the standard treatment approach for patients with advanced prostate cancer. While tumors initially respond to ADT, most eventually progress to CRPC. In a study evaluating the impact of ADT on B7-H3 expression during CRPC progression, a positive correlation between B7-H3 expression and androgen receptor signaling was reported. The presence of androgen receptor binding sites within the B7-H3 gene further supports the notion that B7-H3 expression may be regulated by androgen receptor-mediated mechanisms [4]. Consequently, several studies have proposed B7-H3 inhibition as a promising therapeutic strategy in prostate cancer [8]. In the present study, B7-H3 expression was higher in the pre-CRPC group than in localized disease. Within the pre-CRPC group, B7-H3 exhibited a relatively homogeneous distribution and differed significantly across Grade Groups; however, it did not show statistically robust associations with other clinicopathological variables. These findings are consistent with prior reports linking B7-H3 to aggressive disease features; nevertheless, the near-saturated expression observed in higher-risk groups suggests that B7-H3 should be interpreted not as an independent prognostic marker in isolation, but within a multifactorial framework and in conjunction with other biomarkers.

ISM-1 is an adipose tissue-derived protein involved in multiple biological processes, including glucose and lipid metabolism, and has attracted increasing attention in recent years within the context of tumor biology. Studies have reported that ISM-1 expression may display heterogeneous patterns in both tumor cells and the tumor microenvironment across different solid malignancies [9]. Accordingly, investigations exploring the role of ISM-1 in tumorigenesis have gained prominence in recent years. Experimental studies have demonstrated that ISM-1 can suppress melanoma growth in murine models by inhibiting angiogenesis [13]. In hepatocellular carcinoma, ISM-1 has been shown to inhibit cell proliferation and migration, while in colorectal carcinoma, it has been reported to induce apoptosis and suppress tumor cell proliferation [14,15]. Conversely, increased ISM-1 expression has been described in colorectal carcinoma, adrenal tumors, and adenoid cystic carcinoma [16,17]. Another study suggested that ISM-1 may serve as a prognostic marker in lobular breast carcinoma and may be associated with improved clinical outcomes [18]. In contrast, a study focusing on invasive ductal carcinoma of the breast demonstrated significantly reduced ISM-1 expression in tumor tissue compared with normal breast tissue [19].

Although ISM-1 is known to play a role in cancer biology, the underlying molecular mechanisms remain incompletely understood. In one mechanistic study, interactions between ISM-1, epidermal growth factor receptor (EGFR), and Y-box binding protein 1 (YBX1) were shown to promote colorectal cancer growth, and ISM-1 expression was proposed to be regulated by hypoxia-inducible factor 1-alpha (HIF-1α). In that study, high ISM-1 expression was associated with unfavorable prognostic features [20]. Furthermore, elevated ISM-1 expression in colorectal carcinoma has been linked to reduced overall survival and identified as an independent prognostic factor [16]. When prostate cancer hypoxia is considered within the HIF-1α axis, it may become particularly relevant under the selective pressure imposed by ADT. However, the increased ISM-1 expression observed in pre-treatment diagnostic biopsies from patients who subsequently developed CRPC may reflect a pre-existing stress-responsive state rather than a direct effect of therapy. This interpretation is hypothesis-generating and warrants functional validation of HIF-1α-linked regulation in prostate cancer. Collectively, these findings indicate that ISM-1 may exert context-dependent and multifaceted roles in cancer biology.

Despite this emerging evidence, the role of ISM-1 in prostate cancer—and particularly in the context of patients who subsequently develop CRPC—has not been systematically investigated to date. ISM-1 is an adipose tissue-derived secreted protein; however, immunohistochemical studies have reported that staining in tumor tissues may be heterogeneous at the cellular level [11,19]. To the best of our knowledge, the present study represents one of the first comprehensive immunohistochemical evaluations of ISM-1 expression in this clinical setting, thereby addressing a significant gap in the existing literature.

In our pre-CRPC group, ISM-1 expression was higher than in LPC. However, within the pre-CRPC group, ISM-1 expression showed no statistically significant associations with most clinicopathological variables. In contrast, ISM-1 H-scores differed significantly across Grade Groups, suggesting that its expression may reflect grade group-related tumor biology. These findings indicate that ISM-1 may not function as a direct marker of intrinsic tumor aggressiveness independent of grade. Notably, in time-to-event analyses, the observation of a non-significant trend toward a lower risk of developing castration resistance (HR < 1) with higher ISM-1 expression suggests a potential association between ISM-1 expression and the tempo of progression under ADT. This pattern raises the possibility that elevated ISM-1 expression may characterize a biologically distinct subgroup with a more protracted course to treatment resistance, rather than an aggressive clinical course defined by early and rapid resistance development. Nevertheless, as this association did not reach statistical significance, these findings should be interpreted within a descriptive and hypothesis-generating framework rather than as evidence of causality.

In the present study, the strong and positive correlation identified between ISM-1 and B7-H3 H-scores in the pre-CRPC group indicates that these two molecules—despite their distinct biological functions—may exhibit concurrent and co-varying expression patterns within tumor biology. While B7-H3 has emerged primarily as a marker reflecting tumor aggressiveness and invasive behavior, ISM-1 may be more closely associated with the tempo of progression under ADT, rather than intrinsic aggressiveness per se. Taken together, the concurrent evaluation of these markers suggests that progression to castration resistance cannot be adequately explained within a single-dimensional framework of tumor aggressiveness alone. This integrative perspective supports the concept that patients who subsequently develop CRPC may comprise biologically distinct subgroups characterized not only by aggressive behavior but also by differential capacities for progression under androgen deprivation. From this standpoint, the combined assessment of ISM-1 and B7-H3 may provide a conceptual foundation for future studies aimed at refining patient stratification and management in patients at risk of developing CRPC. Although the present findings are insufficient to support direct clinical implementation, the joint consideration of these two biomarkers may contribute to a more comprehensive and multidimensional understanding of disease biology in this clinical setting.

In prostate cancer, PSA remains the traditional serum biomarker for diagnosis and monitoring [21]. In our study, serum PSA was markedly higher in the pre-CRPC group than in the localized group (median 144.50 vs. 6.48). This indicates substantial baseline imbalance and a potential risk of confounding in between-group comparisons. Therefore, ISM-1 and B7-H3 should be considered tissue-based biomarkers that may provide complementary biological information beyond PSA kinetics, alongside other proposed markers such as AR pathway activity, proliferation markers, imaging targets (e.g., PSMA), and immune-related molecules. In addition, the absence of a correlation between ISM-1 H-score and PSA within the pre-CRPC cohort (Spearman r = −0.038) suggests that the observed ISM-1 signal cannot be explained solely by PSA levels; however, we believe that these findings require confirmation in larger, prospectively designed studies.

This study has several limitations that should be taken into account when interpreting the findings. First, the retrospective design and the small number of cases in certain subgroups reduce statistical power and increase the likelihood of type II error, potentially obscuring true associations. Second, our pre-CRPC group consists of diagnostic tru-cut biopsy specimens obtained prior to ADT from patients who subsequently developed CRPC. Therefore, inferences regarding the relationship of these biomarkers to “therapy adaptation” or “resistance biology” should be viewed not as causal evidence but as hypothesis-generating observations that may reflect baseline biological differences associated with the subsequent clinical course. The marked imbalance in Grade Group distribution between groups, together with differences in tissue type (prostatectomy specimens in localized cases versus biopsy tissue in the pre-CRPC group), further complicates attribution of observed differences to resistance development per se rather than grade-related tumor biology. In addition, the near-ceiling B7-H3 staining pattern in the higher-risk group may have constrained the biomarker’s dynamic range, limiting the ability to detect associations with clinicopathological parameters. Owing to sample size limitations and between-group imbalances, the scope of multivariable adjustment was restricted; divergence in clinical variables such as age and PSA also hindered full control of potential confounding. Finally, without functional/molecular validation, mechanistic interpretation remains limited; accordingly, these descriptive findings should be confirmed in larger prospective cohorts.

## 5. Conclusions

In conclusion, this study sheds light on a previously underexplored biological dimension of ISM-1 in pre-treatment diagnostic biopsies obtained prior to ADT from patients who subsequently developed CRPC, and demonstrates that ISM-1 shows a strong co-variation with B7-H3. When evaluated together, these two markers may help conceptually frame baseline tumor biology associated with higher-grade disease as well as differences in the clinical trajectory toward castration resistance. Overall, our findings support further investigation of ISM-1 and its potential contribution to prostate cancer biology when interpreted in conjunction with B7-H3.

## Figures and Tables

**Table 1:** Comparison of demographic, clinical, and immunohistochemical characteristics between pre-CRPC and LPC groups.

Variables	Pre-CRPC (*n* = 32) Median (Q1–Q3)	LPC (*n* = 30) Median (Q1–Q3)	*p*
Age (years)	72.50	64.50	**<0.001**
	(67.25–80.50)	(60.00–68.25)	
Serum PSA level (ng/mL)	144.50	6.48	**<0.001**
	(92.65–338.75)	(5.18–8.19)	
ISM-1 H-score	6.00	0.00	**<0.001**
	(1.00–9.00)	(0.00–0.00)	
B7-H3 H-score	9.00	6.00	**<0.001**
	(9.00–9.00)	(3.00–6.00)	
Comorbidity, *n* (%)	24 (75)	15 (50)	**0.042**

The LPC group represents localized prostate cancer cases treated with radical prostatectomy, whereas the pre-CRPC group represents cases that subsequently developed CRPC and were diagnosed prior to initiation of ADT. Statistical significance was defined as *p* < 0.05.

**Table 2:** Clinical and pathological characteristics of patients in the pre-CRPC group.

Variables	*n*	%
Grade Group		
2	2	6.3
3	4	12.5
4	9	28.1
5	17	53.1
Seminal Vesicle Invasion		
Present	14	43.8
Absent	18	56.3
Perineural Invasion		
Present	23	71.9
Absent	9	28.1
Lymph Node Metastasis		
Present	27	84.4
Absent	5	15.6
Bone Metastasis		
Present	27	84.4
Absent	5	15.6
Radiotherapy		
Applied	9	28.1
Not applied	23	71.9
Chemotherapy		
Applied	13	40.6
Not applied	19	59.4
Time to Castration Resistance (months)		
Median (25–75th percentile, IQR)	12.00 (9–21)	

Time to development of castration resistance was defined as the interval from the date of initial pathological diagnosis to the time at which castration resistance criteria were first met under androgen deprivation therapy.

**Table 3:** Associations between ISM-1 and B7-H3 H score and clinical and pathological variables in the pre-CRPC group.

Variables	Category	ISM-1 H Score			*p*	Cliff’s δ	B7-H3 H Score			*p*	Cliff’s δ
		M	25%	75%			M	25%	75%		
Grade Group ^a^	2	5	1	9	<0.001	-	9	9	9	<0.001	-
	3	9	8	9			9	9	9		
	4	2	1	6			9	7.5	9		
	5	6	2	9			9	9	9		
Comorbidity	Present	6	2	9	0.717	−0.089	9	9	9	0.999	0.005
	Absent	6	1	9			9	9	9		
Seminal Vesicle Invasion	Present	3	1	9	0.135	0.313	9	9	9	0.925	0.024
	Absent	8	2	9			9	9	9		
Perineural Invasion	Present	9	1	9	0.363	−0.217	9	9	9	0.536	−0.145
	Absent	2	2	6			9	9	9		
Lymph Node Metastasis	Present	6	1	9	0.122	0.452	9	9	9	0.614	0.148
	Absent	9	9	9			9	9	9		
Bone Metastasis	Present	6	1	9	0.448	0.222	9	9	9	0.725	−0.111
	Absent	9	6	9			9	9	9		
Radiotherapy	Applied	2	1	9	0.536	0.150	9	9	9	0.934	−0.024
	Not applied	6	2	9			9	9	9		
Chemotherapy	Applied	6	2	9	0.999	0.001	9	9	9	0.323	−0.211
	Not applied	6	1	9			9	9	9		

^a^: ISM-1 H score: *p*_2–3_ = 0.001; *p*_2–4_ = 0.003; *p*_2–5_ < 0.001, B7-H3 H score: *p*_2–3_ = 0.015; *p*_2–4_ = 0.003; *p*_2–5_ < 0.001. The results were calculated using Dunn’s test. Statistical significance was defined as *p* < 0.05.

**Table 4:** Cox Proportional Hazards Regression Results.

Omnibus Tests of Model Coefficients		−2 Log Likelihood	164,273
	Overall (score)	Chi-square	1.383
		df	2
		*p*	0.501
	Change From Previous Step	Chi-square	1.323
		df	2
		*p*	0.516
	Change From Previous Block	Chi-square	1.323
		df	2
		*p*	0.516
Variables in the Equation		ISM-1 H score	B7-H3 H score
	B	−0.063	0.061
	SE	0.054	0.119
	Wald	1.355	0.261
	*p*	0.244	0.609
	Exp (B) (Cl)	0.939 (0.845–1.044)	1.063 (0.841–1.343)

Event: development of castration resistance. Time was defined as the interval from diagnosis to development of castration resistance (months). ISM-1 and B7-H3 H-scores were entered into the model as continuous variables. B, regression coefficient; SE, standard error; Wald, Wald test statistic; Exp (B), hazard ratio (HR); CI, 95% confidence interval; *p*, two-sided *p* value. HR < 1 indicates a lower risk, whereas HR > 1 indicates a higher risk of developing castration resistance. Statistical significance was defined as *p* < 0.05.

**Table 5:** Correlation analysis between ISM-1 and B7-H3 H-scores in the pre-CRPC group.

Variables	Spearman’s ρ (rho)	*p*
ISM-1 H-score vs. B7-H3 H-score	0.662	<0.001

A *p*-value < 0.05 was considered statistically significant.

## Data Availability

The original contributions presented in this study are included in the article. Further inquiries can be directed to the corresponding author.

## Abbreviations

The following abbreviations are used in this manuscript:

ISM-1	Isthmin-1
B7-H3 (CD276)	B7 homolog 3
LPC	Localized Prostate Cancer
CRPC	Castration-Resistant Prostate Cancer
Pre-CRPC	Pre-treatment diagnostic biopsy tissue from patients who subsequently developed Castration-Resistant Prostate Cancer
PSA	Prostate-Specific Antigen
ADT	Androgen Deprivation Therapy
PCa	Prostate cancer
PNI	Perineural invasion
SVI	Seminal vesicle invasion
LNM	Lymph node metastasis
BM	Bone metastasis
